# Using drone-retrieved multispectral data for phenomic selection in potato breeding

**DOI:** 10.1007/s00122-024-04567-3

**Published:** 2024-03-06

**Authors:** Alessio Maggiorelli, Nadia Baig, Vanessa Prigge, Julien Bruckmüller, Benjamin Stich

**Affiliations:** 1 Institute of Quantitative Genetics and Genomics of Plants (QGGP), Heinrich-Heine-University, Universitätsstraße 1, 40225 Düsseldorf, Germany; 2 SaKa Pflanzenzucht GmbH & Co. KG, Eichenallee 9, 24340 Windeby, Germany; 3 Cluster of Excellence on Plant Sciences (CEPLAS), Heinrich-Heine-University, Universitätsstraße 1, 40225 Düsseldorf, Germany; 4 Julius Kühn-Institut (JKI), Institute for Breeding Research on Agricultural Crops, Rudolf-Schick-Platz 3a, 18190 Sanitz, Germany

## Abstract

Predictive breeding approaches, like phenomic or genomic selection, have the potential to increase the selection gain for potato breeding programs which are characterized by very large numbers of entries in early stages and the availability of very few tubers per entry in these stages. The objectives of this study were to (i) explore the capabilities of phenomic prediction based on drone-derived multispectral reflectance data in potato breeding by testing different prediction scenarios on a diverse panel of tetraploid potato material from all market segments and considering a broad range of traits, (ii) compare the performance of phenomic and genomic predictions, and (iii) assess the predictive power of mixed relationship matrices utilizing weighted SNP array and multispectral reflectance data. Predictive abilities of phenomic prediction scenarios varied greatly within a range of − 0.15 and 0.88 and were strongly dependent on the environment, predicted trait, and considered prediction scenario. We observed high predictive abilities with phenomic prediction for yield (0.45), maturity (0.88), foliage development (0.73), and emergence (0.73), while all other traits achieved higher predictive ability with genomic compared to phenomic prediction. When a mixed relationship matrix was used for prediction, higher predictive abilities were observed for 20 out of 22 traits, showcasing that phenomic and genomic data contained complementary information. We see the main application of phenomic selection in potato breeding programs to allow for the use of the principle of predictive breeding in the pot seedling or single hill stage where genotyping is not recommended due to high costs.

**Supplementary Information:**

The online version contains supplementary material available at 10.1007/s00122-024-04567-3.

## Introduction

To reach the sustainable developmental goal of “zero hunger” as declared by the United Nations (United Nations General Assembly 2015), humanity has to solve complex challenges related to the production and distribution of food (United Nations General Assembly 2015). As one of the top five crops of the world in terms of production quantity and a major source of carbohydrates (FAOSTAT database 2022), potato (*Solanum tuberosum* L.) plays a critical role for food security, especially since its production and consumption constantly increases in developing countries (Devaux et al. 2021; FAOSTAT database 2022). Unfortunately, the genetic gain in potato is not as high as for other species and potato breeders face multiple challenges in developing new varieties, including (i) the tetraploidy and high heterozygosity of the plant (Jansky et al. 2016), (ii) multiple different market segments demanding a high number of traits to be balanced out simultaneously (Tiemens-Hulscher et al. 2013, p.13), and (iii) the fact that important traits can only be assessed very late in the breeding program (Bradshaw 2017).

For most of potato breeding history, plant breeders relied on recurrent phenotypic selection to choose good candidates for growing in the next seasons (Jansky and Spooner 2018). While this approach can be sufficient for simple traits, selection for complex traits, where a myriad of genes and their interactions can have varying influences on the phenotype, poses as a significantly harder challenge. New approaches to identify important genes and their contribution to traits of interest emerged after genetic markers in the form of single nucleotide polymorphisms (SNP) became more and more available (Uitdewilligen et al. 2013; D’hoop et al. 2014). Marker-assisted selection (MAS) in particular has been reported to have the potential to reduce the time of a typical potato breeding program from ten to four years (Slater et al. 2014). However, the accuracy of MAS for complex traits is limited, since it only considers the variance from a limited number of significant quantitative trait loci (QTLs) which in turn usually explain a low proportion of trait variance each (Slater et al. 2016). This problem is overcome by genomic selection (GS), which has been proposed by Meuwissen et al. (2001). In contrast to MAS, GS considers all markers jointly without prior significance testing and, thus, assumes that all markers are potentially linked to the genes affecting the trait of interest. Originally developed in the context of animal breeding, GS found its way into plant breeding quickly and is becoming a state-of-the-art method for selection (for review see Jannink et al. 2010; Crossa et al. 2017; Voss-Fels et al. 2019). The efficacy of GS to predict phenotypic performance has been investigated for potato with promising results (e.g., Slater et al. 2016; Stich and Van Inghelandt 2018). However, the integration of GS in the first stages of typical potato breeding programs is hindered by the high costs for genotyping due to the very high numbers of clones available in these stages (Stich and Van Inghelandt 2018; Wu et al. 2023).

Phenomic selection (PS) was recently proposed as a high-throughput and less expensive alternative to GS (Rincent et al. 2018). The underlying assumption of PS is that phenomic instead of raw genetic information can be used to infer genetic similarities between genotypes. For this, Rincent et al. (2018) built a relationship matrix derived from measured absorbance spectra of different tissues of wheat and poplar via near-infrared spectroscopy (NIRS), and used it in prediction models. The comparative performance of phenomic and genomic prediction in terms of their predictive ability (PA) was dependent on the tissue, the trait, the treatment, and the species considered. In some cases, phenomic prediction outperformed genomic prediction even if the NIRS data used to build the relationship matrix were from a different environment than the calibration set used to train the model (Rincent et al. 2018). Robert et al. (2022a) described phenomic prediction as a “black-box” method since the underlying biological connection between genetics and the reflectance of a given wavelength is complex and harder to interpret biologically than for other endophenotypes. Nevertheless, several studies have emerged using spectral data as a proxy for genetic relatedness since its proof of concept. Some of these capitalized on recent technological advancements and used an unmanned aerial vehicle (UAV) equipped with a hyperspectral camera instead of NIRS to collect reflectance measures for phenomic predictions and also reported high PAs (Krause et al. 2019; Galán et al. 2020). Utilizing a drone to gather reflectance data significantly reduces work effort and time and is thus of interest to breeders. Krause et al. (2019) and Galán et al. (2020) considered a smaller range of wavelengths derived from hyperspectral imaging in contrast to phenomic prediction studies utilizing NIRS. However, it has not been tested yet to what extent a sparse spectrum yields accurate predictions, in addition to a narrow range spectrum. Moreover, phenomic predictions have predominantly been tested with cereal species, raising the question of how the concept of phenomic prediction extends to other crops crucial for food production.

The objectives of this study were to (i) explore the capabilities of phenomic prediction based on drone-derived multispectral reflectance data in potato breeding by testing five different prediction scenarios on a diverse panel of tetraploid potato material from all market segments and considering a broad range of traits, (ii) compare the performances of phenomic and genomic predictions, and (iii) assess the predictive power of relationship matrices utilizing weighted SNP array and multispectral reflectance data simultaneously.

## Materials and methods

### Plant material and experimental design

Our study was based on 466 tetraploid *Solanum tuberosum* L. clones provided and grown by SaKa Pflanzenzucht GmbH & Co. KG (Windeby, Germany). The material consisted of 458 clones of the A clone level (Table 1) which is the first stage in a typical potato breeding program in which more than one tuber can be tested but where the number of tubers per clone is not high enough to test the material in multiple environment trials (METs) (Table 1). The A clones were randomly chosen from SaKa’s breeding program representing multiple market-purpose groups. Eight clones of the set were elite potato cultivars used as comparative checks. These were selected to represent different maturity groups and were also selected from a broad range of market-purpose groups. In total, the chosen A clones belonged to 107 families with an average of four clones per full-sib family. The largest family comprised 38 clones. All clones were grown in two different locations in Germany, namely Windeby (W, Schleswig-Holstein) and Gransebieth (G, Mecklenburg Western Pomerania), and in three different years: 2019 (W), 2020 (W & G), and 2021 (W & G) resulting in five different year and location combinations which were designated in the following as environments.

**Table 1:** Standard potato breeding scheme and dimensioning (Stich and Van Inghelandt 2018)

Year	Stage/activity	No. of clones	No. of tubers per clone in trials and multiplication
1	Cross		
2	Pot seedling	140 000	1
3	Single Hill	90 000	1
4	A clone	5 000	10
5	B clone	600	60
6	C clone	100	300
7	D clone	30	1 200
8	Official trials 1	8	6 000
9	Official trials 2	4	20 000

The clones were grown in augmented row-column designs with one replicate per clone per environment except for the eight checks that were replicated eight times each. Each check was present once per block, resulting in eight blocks per environment. The experiments at both environments in 2021 were separated into two trials representing two different maturity groups: extra early + early (with three blocks) and medium early + medium late (with five blocks). Plots consisted of one row with ten plants in 2019 and two rows with eight plants per row in 2020 and 2021. Plants were phenotyped by SaKa during the growing season (e.g., plant emergence, maturity, foliage development, etc.) and after harvesting (e.g., starch content, tuber length, tuber yield, etc.) for 22 traits (Table S2). The assessed traits included symptoms of disease (rhizoctonia symptoms, scab symptoms), developmental criteria (maturity, foilage development, emergence), measured traits (yield, starch content, PPO), as well as tuber specific quality traits (shape, eye depth, fractions of certain sizes, skin type, etc.). In detail, yield was measured as kg per single plant, calculated by the total yield of a plot in kg divided by the number of grown plants in it (Table S2). All experiments were conducted by SaKa using local agronomical management practices.

### Multispectral data

Multispectral data were obtained for W20 and W21 by overflight with an UAV of model XR6 Hexacopter by Air6 Systems. The drone was equipped with a Tetracam MicroMCA camera with six channels, two of which measured absorbance at near-infrared level, and took pictures at an altitude of approximately 100 ms above the field. Raw images were (i) calibrated and coregistered with PixelWrench (Tetracam Inc.), (ii) photogrammetrically evaluated with Metashape (Agisoft), and lastly (iii) the plot values were statistically assigned with MiniGIS 2.0 (geo-konzept GmbH). Absorbances were measured per plot where the outer sides of 20–30 cms width were not included to minimize edge and soil effects. For 2020 and 2021, different cameras of the same model but with different available channels were used, resulting in multispectral data with five overlapping and two different channels across both years (Table S1). Due to a malfunction, the 670nm channel was not assessed in 2021.

The UAV was used to take pictures on three flight dates in both years. The flight dates were chosen at specific phenological growth stages according to the BBCH scale (Meier et al. 1997), where the two medium-early maturing check varieties, Agria and Verdi, were chosen as reference. The first flight took place at approximately BBCH scale stage 31 (main stem elongation stage with beginning crop cover), the second at approximately stage 65 (first inflorescence flowering stage), and the last flight was performed at approximately stage 91 (beginning senescence) in both years. Multispectral data were gathered and preprocessed by geo-concept GmbH. We used the mean reflectances per plot for each channel and flight date combination for our analyses. Spectral data were scaled, centered, and evaluated for outliers via principal component analysis. We identified 2.8% and 2.6% outliers in the spectra of W20 and W21, respectively, and set them to missing value. Removed outliers were median imputed.

### SNP array

Two Axiom arrays with 947,845 SNPs, described in detail by Baig et al. (unpublished), have been used to genotype the potato clones in our study. The calls were coded in the following way: AAAA = 0, AAAB = 1, AABB = 2, ABBB = 3, and BBBB = 4. The array analysis was performed with Affymetrix GeneTitan system according to the manufacturer’s user guide to get the intensity files (.CEL) which were processed with Axiom Genotyping Algorithm (v.1) (Ax226 iom GT1) in Axiom analysis suite workflow (Nicolazzi et al. 2014). Quality control was carried out following the same Axiom analysis suit workflow. Clones passing array quality score (DQC $\ge$ 0.82) and QC call rate $\ge$ 0.87 were retained for analysis of the SNP genotyping data using R package fitPoly (Voorrips and Gort 2018) by setting the p threshold to 0.95, call threshold to 0.60, and peak threshold to 0.99. SNPs with a call rate of less than 80% were discarded based on cluster plots generated with the R package SNPolisher (Nicolazzi et al. 2014). Furthermore, markers with $\ge 20\%$ missing data and minor allele frequencies below $5\%$ were discarded and the remaining missing values were median imputed leaving 595,321 markers for further analyses.

### Statistical analysis

#### Phenotypic data

To determine the trial effect in the 2021 data, where the material was divided into extra early + early and medium early + medium late maturing clones, we used mixed models using the standard check varieties present in each block similar to Frey et al. (2016) for each trait and each environment separately:

1 $$\begin{aligned} Y_{crbsm} = \mu + M_{ m } + S_{ s } + B_{ b } + R_{ r } + C_{ c } \, + \epsilon _{ crbsm } , \end{aligned}$$

where $Y_{crbsm}$ was the phenotypic observation in the *m*th trial of the *s*th check variety in the *b*th incomplete block, *r*th row and *c*th column, $\mu$ the general mean, $M_{ m }$ the effect of the *m*th trial, $S_{ s }$ the effect of the *s*th check variety, $B_{ b }$ the effect of the *b*th incomplete block, $R_{ r }$ the effect of the *r*th row, $C_{ c }$ the effect of the *c*th column, and $\epsilon _{ crbsm }$ the residual error term. All terms except for $M_{ m }$ were considered random. Phenotypic observations of the checks and entries were then adjusted for each trait by subtracting the trial effect for each clone in the corresponding trial and environment and were used for all following analyses. The trial effect was significant for 9 out of 22 traits but was subtracted for all traits.

Each of the 22 phenotypic traits (Table S2) as well as each of the 21 different flight day $\times$ channel reflectance combinations was analyzed using the following mixed linear model:

2 $$\begin{aligned} Y_{ gcrbe }&= \mu + G_{ g } + E_{ e } + EG_{ ge } + B_{ be }\nonumber \\&\quad + R_{ re } + C_{ ce } + \epsilon _{ gcrbe } , \end{aligned}$$

where $Y_{ gcrbe }$ was the response variable (either trial-adjusted phenotypic observation or scaled and centered channel reflectance) of the *g*th clone in the *b*th incomplete block, *r*th row and *c*th column, nested in the *e*th environment, $G_{ g }$ was the fixed effect of the *g*th clone, $E_{ e }$ the random effect of the *e*th environment, $EG_{ ge }$ the random interaction effect between the *g*th clone and the *e*th environment, and $B_{ be }$, $R_{ re }$ and $C_{ ce }$ the random effects of the *b*th incomplete block, the *r*th row and *c*th column, respectively, all nested in the *e*th environment. Outliers were removed based on visual inspections of quantile–quantile normal plots as well as residuals versus fitted values plots generated with model (2). The trait fraction of small tubers was square root transformed for further analyses.

Every block consisted of up to six columns. To decide if a random column effect in addition to a random block effect was needed, a likelihood-ratio test was used in all models and for all traits testing whether the variance of the random column effect $\sigma _{ c }^2$ is significant (Crainiceanu and Ruppert 2004). If $\sigma _{ c }^2$ was significant, the random column effect was included in the model. All above-described data were preprocessed and analyzed in R version 4.0.2 using custom code (R Core Team 2020). All linear mixed effect models were built with the R package lme4 (Bates et al. 2015) and all likelihood-ratio tests were conducted with the exactRLRT function of the RLRsim R package (Scheipl et al. 2008).

#### Adjusted entry means and broad-sense heritability

Adjusted entry means (AEMs) per clone were calculated for the phenotypic and multispectral data with model (2).

The data collected in each individual environment were analyzed using a simplified version of model (2):

3 $$\begin{aligned} Y_{ gcrb } = \mu + G_{ g } + B_{ b } + R_{ r } + C_{ c } + \epsilon _{ gcrb } , \end{aligned}$$

where the random environmental effect $E_{ e }$ and the random clone $\times$ environment interaction effect $EG_{ ge }$ were discarded from the model. AEMs were also estimated for each clone in each individual environment using model (3) in order to predict phenotypic performance in specific single environments.

Broad-sense heritability for the phenotypic traits was calculated as suggested by Piepho and Möhring (2007) as:

4 $$\begin{aligned} H^{ 2 } = \frac{ \sigma ^{ 2 }_{ g } }{ \sigma ^{ 2 }_{ g } + {\bar{v}} / 2} , \end{aligned}$$

where $\sigma ^{ 2 }_{ g }$ was the genotypic variance and ${\bar{v}}$ was the mean variance of difference between AEMs calculated with model (2). Channel reflectance heritabilities were computed in two ways: (i) for each channel and flight date combination with model (2) and (ii) for each channel by incorporating the flight date *F* and its interactions as random independent variables as a modification of (2):

5 $$\begin{aligned} Y_{ gfcrbe }&= \mu + G_{ g } + E_{ e } + EG_{ ge } + B_{ be }\nonumber \\&\quad + R_{ re } + C_{ ce } + F_{ f } + GF_{ gf } \nonumber \\&\quad + FE_{ fe } + GFE_{ gfe } + BF_{ bf } \nonumber \\&\quad + RF_{ rf } + CF_{ cf } + \epsilon _{ gcrbe }. \end{aligned}$$

For the latter case, heritabilities were also calculated on a plot basis. $\sigma ^{ 2 }_{ g }$ was determined by setting $G_{ g }$ of model (2) or (5) as random.

Since two channels were non-overlapping and one of the overlapping channels malfunctioned for one year, missing data were imputed in these three environment/year combinations via predictive mean matching, which is a semi-parametric imputation approach that produces plausible values (Van Buuren 2018, p.68) with the R package mice (Van Buuren and Groothuis-Oudshoorn 2011).

### Estimation of breeding values

#### Prediction model and calculation of relationship matrices

Phenotypic performance was predicted based on the genomic best linear unbiased prediction (GBLUP) model (VanRaden 2008):

6 $$\begin{aligned} Y_{ u } = \mu + U_{ u } + \epsilon _{ u }, \end{aligned}$$

where $Y_{ u }$ was the AEM and *U* the random genetic effect of the *u*th clone, respectively. *U* was independent of $\epsilon$ with $U \sim N(0,G\sigma ^2_{ U })$. Here, $\sigma ^2_{ U }$ was the variance of the genetic effects and *G* was the additive genomic relationship matrix defined as $G = \frac{ ZZ' }{ m }$, where $Z'$ was the transpose of *Z* which was the feature measurement matrix of dimensions $n \, \times \, m$, where *n* was the number of clones and *m* was the number of molecular markers (*m* = 595,321). For the phenomic prediction scenarios, in which multispectral data were used for predictions (MBLUP), *G* was replaced by the multispectral relationship matrix *M* which was calculated similarly, except that $ZZ'$ was replaced by $SS'$. *S* was the spectral feature matrix of $n \, \times \, m_{ s }$ where $m_{ s }$ was the number of channel and flight date combinations ($m_{ s }$ = 21).

Additionally, we tested if GBLUP predictive abilities (PAs) could be improved by considering dominance and epistatic effects. For this case, the dominance (D) relationship matrix was calculated with the Sommer R package version 3.3 (Covarrubias-Pazaran 2018). We compared three different GBLUPs considering (i) only additive effects (*G*), (ii) additive and dominance effects ($G + D$), and (iii) additive, dominance, and all three first-degree epistatic interaction effects, namely additive $\times$ additive, additive $\times$ dominance, and dominance $\times$ dominance ($G + D + E$). For this, random genetic effect terms and their corresponding variance–covariance matrix were added in model (6) where $G + D$ included two and $G + D + E$ five random terms and relationship matrices.

Predictions were made within a fivefold cross-validation framework with 25 replications. PA was reported as the Pearson’s correlation between observed and predicted adjusted means of the validation set. Due to some unfortunate training and validation set combinations as well as missing data, especially in cases where single environments were predicted, the MBLUP and GBLUP model did not always converge. Therefore, we applied a quality filter to our results based on the ability to make reliable decisions in a real breeding program: prediction results were removed if they had (i) an absolute coefficient of variation $> 150$ or (ii) $> 100$ missing predictions from a total of 5 folds * 25 replicates = 125 predictions per trait and prediction scenario. All predictions were established using the R package Sommer (Covarrubias-Pazaran 2018).

#### Prediction scenarios

**Fig. 1:**
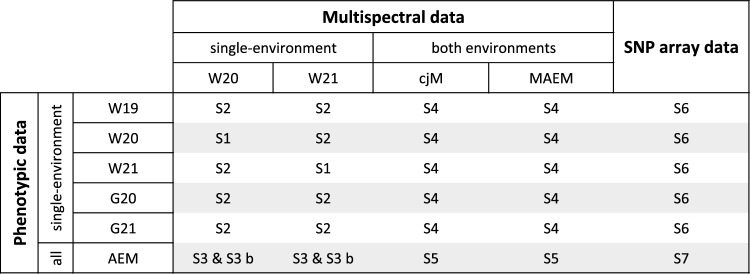
Illustration of the seven prediction scenarios (S1–S7) explored. Each cell represents a prediction case. Each prediction case is a combination of different sources of phenotypic and multispectral/SNP array data used for phenomic or genomic selection with multispectral best linear unbiased prediction (MBLUP) or genomic BLUP (GBLUP), respectively. Predictions were performed based on the adjusted entry mean (AEM) of a given clone in one or multiple environments. Phenotypic data of one of five single environments can be predicted where W and G represent the locations Windeby and Gransebieth, respectively, while 19, 20, and 21 give the year of the experiment. In S3, S5, and S7, phenotypes of clones were predicted based on the AEMs across all five available environments. Additionally, a sub-scenario of S3 (S3 b) was considered where the AEMs did not incorporate the environment from which the spectral data were collected. The relationship matrix used in S4 and S5 was either derived by first joining the spectra of both environments into one (cjM) or by calculating the AEMs of the channel reflectances per clone based on data of both environments (MAEM)

The scenarios to explore phenomic and genomic prediction in potato breeding were the following (Fig. 1):

*Scenario 1* Predictions of phenotypic AEMs of single environments derived with model (3) were made using relationship matrices calculated with multispectral AEMs from model (3) from the same environment, namely W20 and W21 (Fig. 1).

*Scenario 2* This scenario differs from scenario 1 in that predictions were made based on multispectral data collected in single environments for which the phenotypic data have not been collected.

*Scenario 3* AEMs of every trait per clone across all five environments were calculated with model (2) and then predicted with spectra from individual environments (W20 or W21).

*Scenario 3 b* Additionally, a sub-scenario of scenario 3 was investigated in which the phenotypic AEMs did not include the environment from which the spectral data were collected.

*Scenario 4* Multispectral data of both environments were considered for predictions of phenotypes of clones in every available single-environment. The multispectral data were summarized either by using the adjusted means of the channel absorbances per clone from model (2) (S4 MAEM) or simple column-joining of both multispectral data sets (S4 cjM) before building the relationship matrix M (Rincent et al. 2018).

*Scenario 5* In this scenario, the relationship matrices from scenario 4 were used to predict the phenotypic AEMs calculated across all environments.

*Scenario 6* A classical genomic prediction approach was tested using SNP array information to calculate genomic estimated breeding values (GEBVs) for each clone and trait. Predictions were made for every available single-environment.

*Scenario 7* GBLUP was used to predict the phenotypic performance of clones across all environments, i.e., the AEMs derived from model (2).

In addition to predicting phenotypic performance by utilizing *M* or *G* only, we also examined the predictive ability of the model if both relationship matrices were incorporated simultaneously. One possible approach for this purpose was reported by Robert et al. (2022b) who combined the NIR effect and the molecular marker effect with two separate respective relationship matrices in one model. Another solution that produces equivalent PA is a combination of relationship matrices (*C*) by assigning weights to *M* and *G* in a grid search similar to Schrag et al. (2018) and Wu et al. (2022). We investigated a combination of *M* and *G* in the following way:

7 $$\begin{aligned} C = M \times x + G \times (1 - x), \end{aligned}$$

where *x* varied from 0 to 1 in varying step sizes. We then used all variations of C as the relationship matrix in model (6).

## Results

### Trait and channel reflectance heritabilities

Trait heritabilities on an entry mean basis ranged from 0.30 (shape short axis) to 0.93 (starch content) where heritabilities $\ge 0.7$ were observed for 15 out of 22 traits (Table S3). The median proportion of variance accounted by error was 0.11, suggesting that model (2) was a good fit for most traits. In general, traits that were rated on a categorical scale like texture, taste, and general impression had higher error terms and, thus, lower heritabilities than traits that were measured like polyphenol oxidase activity, starch content, and yield.

Channel reflectance heritabilities across flight dates derived with model (5) were low with a median of 0.18 (Table S4). Considering each flight date and channel combination separately in model (2) resulted in significantly higher clone and clone $\times$ environment interaction ($G \times E$) variances and in an increase of the median heritability to 0.39 (Table S3). Model (5) yielded high flight date $\times$ clone and flight date $\times$ clone $\times$ environment effects (Table S4). This trend can also be observed from the variance components of model (2) where $G \times E$ variance varied highly across the flight dates (Table S3).

### Predictive abilities of phenomic prediction scenarios

**Fig. 2:**
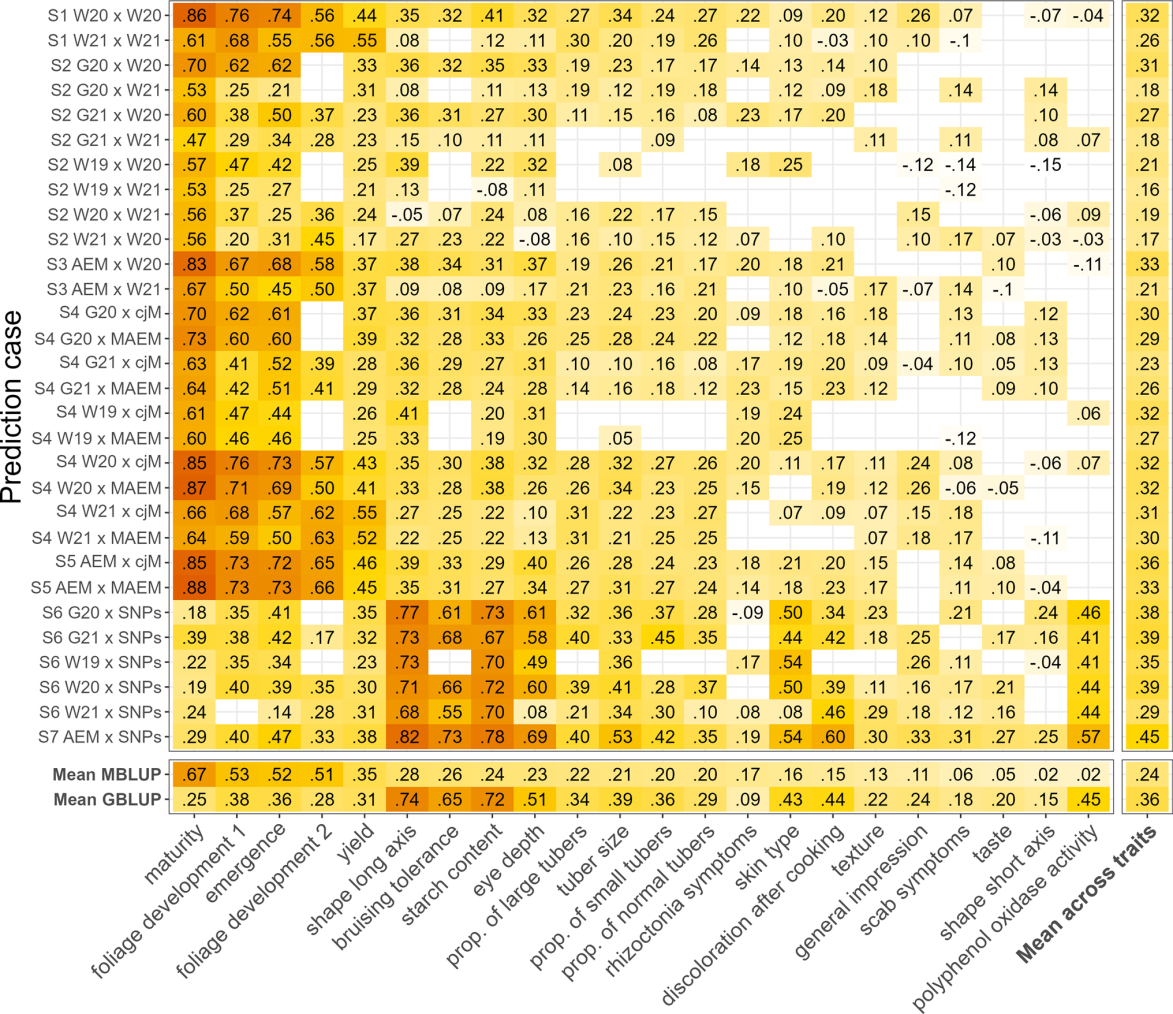
Predictive abilities (PAs) for every potato trait in every prediction case. The median PA is depicted in each tile as a decimal number and is a result of a fivefold cross-validated prediction with 25 repetitions either with multispectral best linear unbiased prediction (MBLUP) for scenarios one to five (S1–S5) or with genomic BLUP (GBLUP) for scenario six and seven (S6 & S7). Traits are ordered on the x-axis according to the mean PA of phenomic prediction scenarios (S1–S5) in descending order. The Y axis shows the prediction case. Each prediction case shows the general prediction scenario (S1–S7). The code before the x specifies which phenotypic data is predicted and the one after the x states from which data the relationship matrix was derived. G and W are the code for the locations Gransebieth and Windeby, respectively, while 19, 20, and 21 represent the year. AEM before the x means that the phenotypic adjusted entry means (AEMs) derived from all five available environments were predicted while MAEM after the x specifies that the AEMs derived from both multispectral data sets, namely W20 and W21, were used for predictions. cjM stands for “column-joined” and means that the two aforementioned matrices of spectra were joined into one single matrix before computing the relationship matrix *M* for prediction. SNPs stands for the GBLUP model where data from a SNP array were used to build the relationship matrix. Missing tiles could either not be calculated due to missing trait data for a specific environment or because the predictions did not pass the quality control of an absolute coefficient of variation $\le 150$ or $\le 100$ missing PAs. Every prediction was carried out using respective relationship matrices only considering additive effects. The mean PA across all traits per prediction case is shown in the last column while the mean PA across each MBLUP (S1–S5) or GBLUP (S6–S7) scenario is shown per trait in the last two rows

The median PAs of phenomic prediction scenarios varied greatly within a range of $-$0.15 and 0.88 and were strongly dependent on the environment, the predicted trait as well as the considered prediction scenario (Fig. 2). Most predicted traits of S1, where phenotypic performance was predicted based on spectral and phenotypic data from the same environment, showed median PAs below 0.3. However, high (0.55) to very high (0.86) PAs were observed for traits that characterize the development of the growing plant above ground and moderately high (0.45–0.55) PAs were achieved for yield (Fig. 2). For few traits like starch content, eye depth, and longitudinal shape, PA was significantly higher for one environment than the other. Comparing the mean PA across all traits showed that S1 W20 $\times$ W20, i.e., when phenotypes of Windeby 2020 were predicted using the relationship matrix derived from Windeby 2020 spectra, achieved, on average, higher PA (0.32) than S1 W21 $\times$ W21 (0.26). PAs in S2, where spectral and phenotypic data used for predictions were collected from different environments, were higher for half of the phenotypic traits compared to S1 when considering the best-performing prediction cases from each scenario and trait combination (Fig. 2). However, it is important to note that the traits for which S2 performed better than S1 were traits for which PAs $\le 0.35$ were observed. S2 PAs also varied highly between the pairs of environments considered for each trait. In total, eight possible S2 prediction cases exist. The S2 prediction case with the highest PAs was G20 $\times$ W20 with an average of 0.31 across all traits. Interestingly, G21 $\times$ W20 and W19 $\times$ W20 were the second and third best-performing prediction cases with a mean PA of 0.27 and 0.21, respectively, which means that predictions with *M* from W20 were in general more accurate than predictions with *M* from W21, if spectra of an independent environment were used.

In the next step, we predicted the AEMs of phenotypic traits across all five environments (S3). For both spectral data sets, 52% of the traits had lower PA and 29% had higher PA in S3 than S1 (Fig. 2). The other traits could not be compared, due to missing median PAs of either one of the two prediction cases. The range of variation of the difference between S3 and S1 was large with an increase of PA of 0.24 for scab symptoms in W21 and a reduction of PA of 0.18 for foliage development also in W21. All traits that performed well in S1 with PAs > 0.4 achieved lower PAs in S3. If the relationship matrix was derived from the spectra of an environment that was not included in the calculation of the AEMs to be predicted for S3 (i.e., S3 b), then a decrease in PA could be observed for 89% of the traits predicted with the relationship matrix from W20 and 53% of traits predicted with the spectra collected in W21 (Fig. S1). Analogous to S1, predictions with *M* from W20 achieved a higher mean PA across all traits (0.33) than predictions with *M* from W21 (0.21).

We evaluated the effect of using spectra from two environments together to predict phenotypes from individual environments (S4). On average across all traits, we observed PAs similar to S1 for these S4 scenarios (Fig. 2). However, S4 performed better than S1 for those traits that showed significantly lower PAs in S1 W21 compared to W20. The S4 scenario comprised two methods to combine spectra from two environments. We observed a slightly higher mean PA across all traits for cjM (0.36) compared to MAEM (0.33) although individual traits deviated from that trend. In the next step, we evaluated the use of spectral data from both environments to predict AEMs across all five environments (S5). This scenario yielded higher PAs than the best-performing environment of S3 for 13 out of 19 traits. In general, the PAs of S5 were on a similar level as the better-performing S1 and S4 prediction cases. Since S5 utilizes every information available and yields high PA, we evaluated the influence of different flight dates on PA using S5. Date one (D1) and its combinations had the biggest positive effect on PA and the flight date combinations D1+D3 and D1+D2+D3 achieved the highest PAs with a median of 0.26 across all traits (Fig. S2). Including data of multiple flight dates in the relationship matrix used for prediction was always significantly favorable except in one case where spectral data from D1 performed similar to data from D2+D3 (Fig. S1).

### Comparison of MBLUP and GBLUP

GBLUP predictions with the additive relationship matrix *G* were made for all five environments independently (S6) as well as for the AEMs across all environments (S7). For the former case, GBLUP performed better than MBLUP for 15 traits in S1, 19 traits in S2, and 16 traits in S4 cjM measured by the aggregated median PA across all available environments per scenario (Fig. 2). In addition, the median absolute coefficient of variation across all single-environment predictions of S6 was lower (25.0) compared to S4 cjM (30.6). Using the SNP array data to predict the AEMs of clones across all five environments (S7) resulted in higher PAs than the median across all five single-environment predictions (S6) for all traits. A similar trend can also be observed in the same comparison between S5 and S4 cjM. However, the difference in this comparison was that the best-performing environment pair of S4 cjM exceeded S5 for 14 traits while the best-performing GBLUP environment pair of S6 exceeded S7 only for four traits.

In addition to the above-described additive GBLUP model (*G*), we tested two more genetic models with S7 (Fig. S3). The additive and dominance ($G + D$) model achieved a median PA of 0.420 across all traits, similar to the PA of the *G* model (0.424). $G + D$ had the highest PAs for five out of 22 traits, namely yield, emergence, shape short axis, tuber size, and starch content while all other traits achieved the highest PA when considering only additive effects (*G*). $G + D + E$ had the lowest PAs with a median of 0.400 across all traits.

**Fig. 3:**
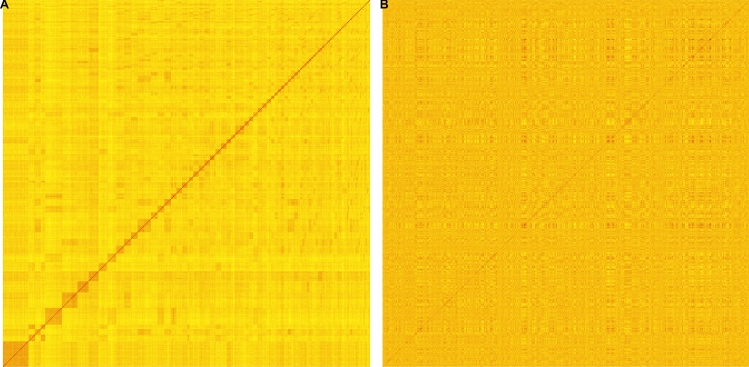
Heatmaps of relationship matrices built either with SNP array data (**A**) or multispectral channel reflectance data (**B**). Both heat maps are sorted according to the family structure of the potato germplasm

When taking a closer look at the relationship matrices used for GBLUP and MBLUP, we observed that the pedigree relatedness of our potato population was represented in *G* (Figs. 3A and S4A). Interestingly, this observation was not made for *M* (Figs. 3B and S4 B). In detail, the Pearson’s correlations between *M* from W20 or W21 and *G* were 0.13 and 0.08, respectively.

### Predictions with weighted relationship matrices

**Fig. 4:**
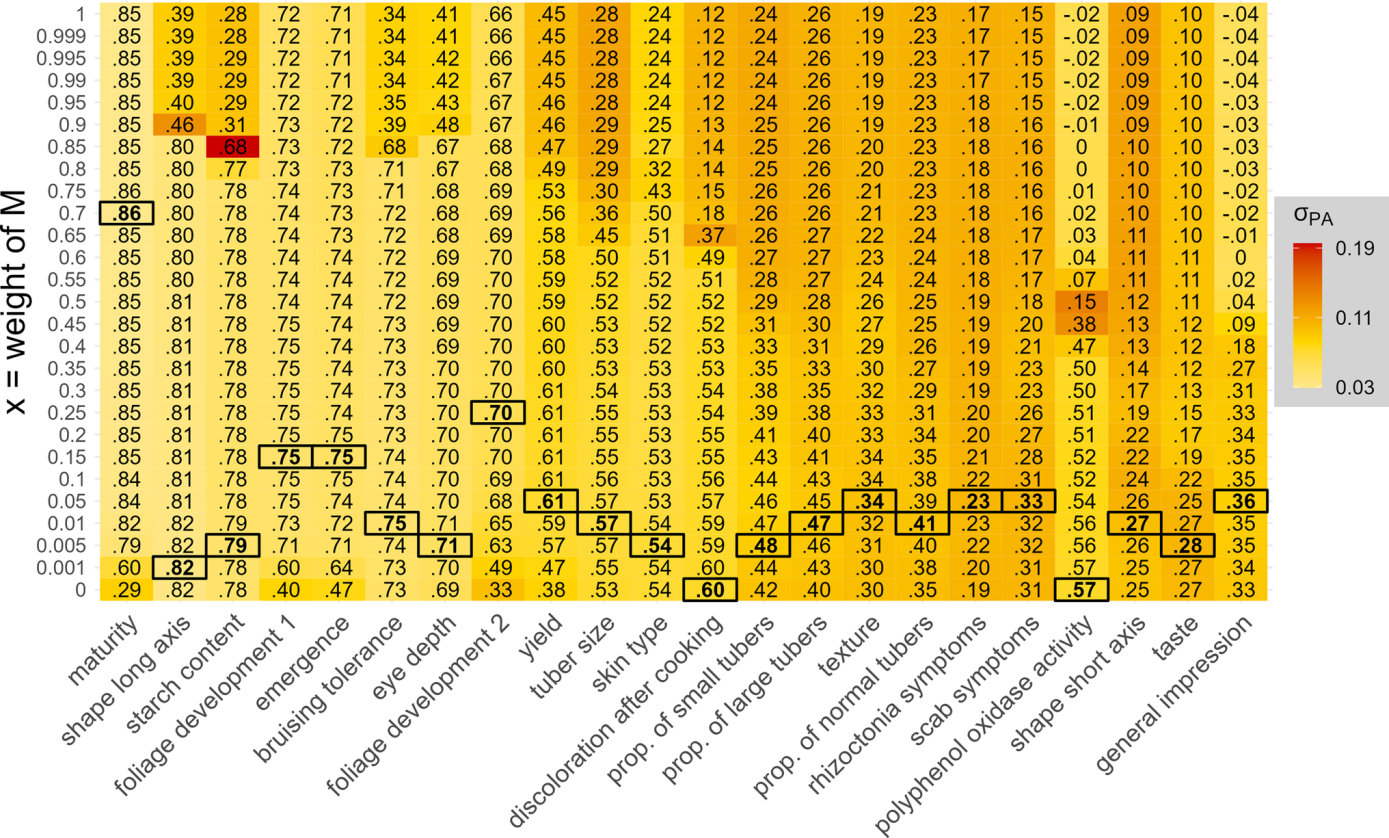
Predictive abilities (PAs) of 22 phenotypic traits with different combinations of weighted relationship matrices. *M* was derived from the multispectral data and *G* was derived from the SNP array data. The y axis shows the weight of *M* (**x**). The weight of *G* was $1-x$. The median PA of a fivefold cross-validated prediction scheme with 25 repetitions represented with each tile is shown as a decimal number. The standard deviation of the respective PAs was shown with colors. For each trait, the highest median PA achieved was marked with a black box

Since the relationship matrices *M* and *G* displayed different similarity patterns among the clones (Figs. 3 and Fig. S4), we tested if a combination of both data sets can lead to an increase in PA (Fig. 4). Interestingly, only two traits, namely, discoloration after cooking and polyphenol oxidase activity, achieved the highest PA by training the prediction model with *G* alone. No trait achieved the highest PA by training the prediction model with *M* alone, and 20 traits scored a maximum PA with a relationship matrix calculated from *M* and *G* together. For four of those aforementioned 20 traits, the PAs were improved notably by $\ge$ 0.05 compared to the maximum of either *M* or *G* alone by combining both matrices for prediction (Fig. 4). The trait that benefitted the most from a combination of *M* and *G* was yield with an increase of 0.16 PA compared to *M* alone and an increase of 0.23 PA compared to *G* alone if *M* was weighted with a factor of 0.05. In general, most maximum PAs observed with mixed relationship matrices were achieved with a higher weight of *G* (*x* $\le$ 0.25) with maturity being the sole exception (*x* = 0.7) (Fig. 4).

## Discussion

### Inferences on genetic relatedness based on UAV imaging data

The basic assumption of phenomic prediction is that reflectance or absorbance measures like NIR spectra are capturing genetic similarities between genotypes (Rincent et al. 2018). Up to now, phenomic prediction has mainly been tested on cereal species (Rincent et al. 2018; Krause et al. 2019; Lane et al. 2020; Galán et al. 2020; Robert et al. 2022b). This is probably due to the routine use of NIRS to predict various features like water and protein content of grains (Pojić and Mastilović 2013), providing access to an already established pipeline for generating phenomic data. However, there is also potential that this approach can be used in potato.

To check in a first step how reliable multispectral channel reflectances are across multiple environments for potato clones, we calculated their variance components and heritabilities (Table S3). Broad-sense heritabilities indicated a low to moderate proportion of reflectance variance explained by genetic variance which means that the reflectances are partly clone-specific. The channel reflectance heritabilities observed for potato were lower than those reported in another study where hyperspectral imaging via UAV was used for phenomic prediction in hybrid rye (Galán et al. 2020). This finding can be explained by the fact that fewer environments were available to calculate heritabilities on an entry mean basis in our case compared to Galán et al. (2020). Other causes ranging foremost from a different species analyzed to a different UAV protocol for generating data as well as different data preprocessing methods can also provide possible explanations.

Channel reflectance heritabilities on a plot basis (Table S4) were higher for the lower wavelengths, meaning that these contributed the most to our predictions. We observed a high variation for genotype-related variance components and heritabilities not only between channels but also between flight dates within channels (Table S3). This means that the ability of multispectral data to capture genetic variance can drastically change with the developmental stage of the plant and for each channel differently. Analyses of the effects of individual flight dates and their combinations on PA revealed that spectra collected at flight date one (D1), i.e., at BBCH stage 31, had on average across all traits the best effect on PA, although individual traits could deviate from that trend (Fig. S2). However, PA was increased if multiple flight dates were combined. Although individual flight dates and their combinations exceeded the PA of D1+D2+D3 for some of the traits, D1+D2+D3 predictions proved to be most robust, i.e., PAs were always on the higher end compared to all other combinations (Fig. S2). Our finding is in agreement with results of Aguate et al. (2017) who also reported an increase in prediction accuracy for maize yield by combining hyperspectral data of multiple time points and considering the reflectance data as secondary traits. More recently, Adak et al. (2023a, b) showed that including drone data collected at multiple growth stages in temporal phenomic prediction models of maize hybrids resulted in increased PA. We, therefore, recommend collecting spectra from several different time points in future studies capitalizing on imaging via UAV. Furthermore, our results suggest that it might be interesting for future experiments to add additional UAV flights around the early potato development stage. More research is needed to decide if it is better for some individual traits, to consider only specific flight date and channel combinations especially, if costs are factored in.

### Phenomic prediction scenarios

We started exploring the capabilities of the MBLUP model to predict phenotypic performance with the relationship matrix *M* that was derived from multispectral data from the same environment for which phenotypic assessments were made (S1). We observed median PAs of 0.44 (W20) and 0.55 (W21) for yield and median PAs between 0.86 and 0.55 for traits that characterize foliage development, emergence, or maturity of the plant. The high PAs were surprising considering the low number of spectral channels available compared to other PS studies using either NIRS (Rincent et al. 2018; Lane et al. 2020; Robert et al. 2022b) or hyperspectral imaging based on UAVs (Krause et al. 2019; Galán et al. 2020). To exclude the possibility that the high PA for yield was due to high correlations with the latter mentioned traits such as maturity or foliage development, we evaluated their correlations. We found significant but low correlations between yield and maturity ($-$0.26) as well as between yield and foliage development (0.33). Furthermore, we applied our S5 cross-validation scheme to a multiple linear regression model that predicts yield by these developmental traits (maturity, emergence, foliage development 1 & 2). Here, the PA was 0.27 as opposed to 0.46 from S5. This illustrates that the reason for the high PA of yield in our phenomic prediction scenario is not only due to its correlation with the easily predicted developmental traits. These findings together strongly reinforce the hypothesis that the spectral data represented endophenotypic information of the potato plant relating to yield but also to many other traits that can be used for phenomic predictions in potato.

In the next step, we tested the performance of MBLUP in predicting the phenotypic performance of clones in one environment if multispectral data of matching clones were only available from a different environment (S2). This represents an interesting approach for breeders since reflectance data could be measured once for a reference site and then used for selection in independent environments. The PAs for S2 were slightly to moderately lower than those observed for S1 (Fig. 2). The decrease of PA from S1 to S2 for yield and emergence was in general more substantial than reported for wheat by Rincent et al. (2018) and Robert et al. (2022b). These observations suggest that predictions of independent environments are generally feasible but are also attached to a loss in PA for potato.

The proportion of variance explained by the clone on the different channel reflectances was very similar to what Rincent et al. (2018) reported for winter wheat leaves. However, the proportion of variance explained by the clone-environment interaction ($G \times E$) effect was higher in our case. This disparity in $G \times E$ might be due to the potato population in our experiment which consisted mostly of clones on the A clone level while Rincent et al. (2018) considered exclusively elite winter wheat varieties which have been selected for broad adaptation.

In the next prediction scenario, we evaluated the effect on PA if spectra from more than one environment were used for prediction (S4 & S5). For S4, we observed similar PAs compared to the best S1 prediction cases (Fig. 2), where a total of 12 traits achieved even slightly higher PA in S4 compared to S1. However, the highest PAs besides S1 and S4 prediction cases were observed for S5 where AEMs across all five environments were predicted utilizing both multispectral data sets like in S4. S5 PAs were higher than both S3 prediction cases, where AEMs were predicted using reflectance data collected from only one environment, for 13 out of 19 comparable traits. The results of S4 and S5 illustrate the value of collecting spectra at multiple environments, especially if the spectra have high $G \times E$ like in potato. These observations are in alignment with the results of previous studies, reporting that the utilization of multiple spectra has an overall positive effect on PA in phenomic predictions (Rincent et al. 2018; Robert et al. 2022b). Our conclusion was further supported by (i) the comparison between S4 and S2, where PAs were generally higher for the former prediction scenario if both spectra were used to predict phenotypes of an independent environment (S4 G20, G21 & W19), and (ii) the fact that if S3 predictions were performed for adjusted clone means which were calculated without the phenotypic data of the environment where the multispectral reflectances were gathered from (S3 b), then the PAs were usually lower than in the original S3 (Fig. S1).

Two possibilities to combine spectral data from different sources (i.e., environments, tissues, flight dates) for phenomic prediction have been reported in the literature. The first method, which we refer to as column-joining (cjM), is the combination of different spectra into one (Rincent et al. 2018) which is equivalent to averaging the relationship matrices from different sources (Lane et al. 2020; Robert et al. 2022a). The second approach is the calculation of channel reflectance AEMs per channel (Galán et al. 2020), in our case, per flight date and across environments. We observed that combining spectra by simple column-joining was slightly favorable (Fig. 2) compared to calculating the reflectance AEMs per clone which could be explained by the fact that deriving AEMs is generally linked with a loss of specificity, while simple column-joining preserves every available measurement.

The most relevant scenarios for potato breeders were examined in S3 and S5: the prediction of AEMs across environments. S3 PAs were higher than individual S2 cases and generally only slightly lower than S1 (Fig. 2). If AEMs across all five environments were predicted using the spectral profiles of both environments (S5), then PAs were higher for 13 out of 19 traits compared to S3 as well as for 10 out of 19 traits compared to S1. This means that S3 and even more so S5 can be understood as robust prediction approaches. They can be used to show the general extent of PA for a set of environments without the danger of considering a particularly badly predicted environment for a given trait.

### GBLUP vs MBLUP performance and PAs using mixed relationship matrices

Genomic prediction is becoming the state-of-the-art method of predictive breeding in many crops (Crossa et al. 2017; Voss-Fels et al. 2019). Therefore, we compared the results of phenomic prediction to those of genomic prediction. We observed that GBLUP performed better than MBLUP for traits characterizing the tuber itself like starch content, eye depth, or polyphenol oxidase activity, while traits that are observable with the eye on the growing plant had high PAs for MBLUP. Yield was an exception to this trend because PAs were higher with phenomic predictions. Zhu et al. (2022) reported for triticale that polygenic traits were well predicted with MBLUP and mono- or oligogenic traits were better predicted with GBLUP. Some of our findings were in accordance with this trend, i.e., the complex trait yield had higher PAs from phenomic predictions while the oligogenic trait polyphenol oxidase activity had higher PAs using GBLUP for example. However, the oligogenic trait maturity (Danan et al. 2011) had higher PAs with phenomic predictions as compared to genomic predictions in our case; thus, this trend was not fully observed in our study.

We observed PAs that varied across environments in S6, where phenotypic performance of clones was predicted in each environment separately using GBLUP. However, the variation in PA across environments in S6 was smaller than that obtained with MBLUP in S4 cjM which is probably due to the moderately high $G \times E$ variance proportion of our spectra (Table S3). Analogous to what we observed for S5, S7 PAs were usually among the highest for GBLUP prediction cases, meaning that the predictions of AEMs across environments were generally more robust than predicting AEMs of a specific environment.

As a highly heterozygous crop, potato allows for dominance as well as epistatic effects to contribute to phenotypic variation. However, our results showed that the GBLUP model that considered additive, dominance, and epistatic effects ($G + D + E$) achieved slightly lower PAs than models that considered additive and dominance ($G + D$) or only additive effects (*G*) (Fig. S3). PA for *G* and $G + D$ were similar, where $G + D$ performed better in predicting the phenotypic performance of yield, emergence, shape short axis, tuber size, and starch content. Thus, depending on the trait and its genetic complexity it can be advisable to model dominance effects with GBLUP when selecting clones for their phenotypic performance which is in accordance with Stich and Van Inghelandt (2018).

The PAs observed with MBLUP and GBLUP indicated that the inferences on relatedness seem to be largely different when derived from the spectral data compared to derived from genetic markers (Fig. 2). This is further supported by the observation of low correlations between the two relationship matrices *M* and *G*, and the fact that the family structure was reflected in the heat map of *G* but not *M* (Fig. 3). Since both approaches achieved high PAs but generally for different traits, we evaluated whether combined relationship matrices could improve PAs. For a total of 20 out of 22 traits, higher PAs were observed if relationship matrix *C* was used for prediction which combined multispectral and SNP array profiles, showcasing that both contain complementary information (Fig. 4). The potential for a combination of marker and spectral data was also demonstrated by Sandhu et al. (2021) and Robert et al. (2022b) where it achieved higher PAs for yield in wheat than genomic or phenomic prediction alone. Similar observations were also made for combined omics data sets like metabolites or transcript levels that resulted in an improvement of the prediction accuracy compared to GS (Schrag et al. 2018; Wu et al. 2022). If the goal is to maximize PA then it seems favorable to include a carefully weighted mix of the aforementioned types of information in predictions. However, considering costs, time, and work effort this might be not practical in real breeding programs. The optimal allocation of resources with the utilization of NIR or other kinds of reflectance measures as a new player in predictive plant breeding, thus, requires further research.

### Integration of PS in potato breeding programs

Current potato breeding programs are characterized by very large numbers of entries in early stages and the availability of very few tubers per entry in these stages (Table 1). Both aspects lead to the need to assess only traits of low genetic complexity that can be scored on individual plants as well as traits that can be measured non-destructively. Many of such traits are only weakly correlated with the traits determining market success (Thelen et al. (unpublished)). In this context, we envisage two possible applications for PS in potato breeding programs.

The first interesting possibility for the integration of PS in potato breeding programs is in the early stages, i.e., pot seedling and/or single hill stage (Table 1), where results from computer simulations suggested that the use of genomic predictions is not recommended due to the high costs for genotyping and the associated strong need to reduce the population size (Wu et al. 2023). Here, PS can be the low-cost alternative to GS to increase the selection accuracy by selecting based on the target trait instead of an auxiliary trait of pot seedlings or potatoes in the single hill stage. One could calibrate MBLUP with the phenotypic data, preferably AEMs, of clones that are at the A clone stage or later from other breeding cycles and use the spectral profiles of not only the corresponding clones but also completely unphenotyped clones in the pot seedling or single hill stage to establish a relationship matrix between all clones in this early stage. This approach is related to S3 b in this study, where the spectral profile of an independent environment is used to predict the phenotypic performance of clones on an AEM basis. An assumption of this approach is that a clone in the pot seedling or single hill stage has a similar spectral profile as in the later stages, i.e., when grown in plots with several plants per clone. Additional investigation is necessary to substantiate this assumption. A further limitation of the prediction from individual single potato plants is the environment specificity of the multispectral profiles as we observed varying PAs depending on the source of the spectra in our study. S4 cjM revealed that it is likely beneficial to combine spectra of multiple environments to buffer against the high $G \times E$ of the spectra in potato. As clones in the pot seedling or single hill stage can only be evaluated in one environment this will likely not be practicable at this stage. However, if predictions of sufficient predictive ability are still feasible in this way, then a considerable increase in selection accuracy can be realized by PS compared to phenotypic selection.

The second possibility to integrate PS in potato breeding programs is in later cycles (A to D in Table 1) when phenotypic information is available for each clone. In these stages, one could either select clones based on their per-se performance as commercial products or parents of new crosses. In the former case, current potato breeding programs largely rely on phenotypic selection which means that the comparative performance between phenotypic selection and PS would depend on (i) the heritability of the target trait, (ii) the heritability of the channel reflectances, (iii) the PA achieved with phenomic predictions, and (iv) the cost of the assessment. Regarding the selection of parents for new crosses, it is advisable to not select based on the true genetic value of a clone since only a third of the digenic dominance is transmitted to the next generation in tetraploids (Gallais 2003; Endelman et al. 2018). However, phenomic predictions based on spectral profiles likely also include non-additive effects (Rincent et al. 2018). If this is the case, then one needs to be cautious in using PS in the selection of parents for new crosses. If spectral profiles can be separated in additive and non-additive effects, a weighting of both might be feasible to select for crossing candidates where the weight depends on the genetic architecture of the trait considered. Both possible implementations represent interesting possibilities for breeders and warrant further research.

## Conclusions

This study establishes a proof of concept of phenomic predictions with potato. PAs of phenomic predictions were high for yield and traits that characterize the development of the plant above ground while genomic predictions achieved higher PAs for traits regarding the tuber itself except yield. High PAs were achieved despite a total of only seven wavelengths available as measured by an UAV. High variability was found in PAs between predicted environments, traits, and multispectral data sets. Our results indicate that combining spectra from different environments to predict phenotypes in the least stabilizes, if not increases PA and, thus, buffers against low-performing environments. Predictions of AEMs across multiple environments also proved robust. Therewith, PS presents a low-cost and high-throughput alternative to GS but is also highly effective in combination as relationship matrices derived from SNP array data and spectral profiles together yielded even higher PAs for 20 out of 22 considered potato traits than either phenomic or genomic prediction alone. We see the main application of PS in potato breeding programs to allow for the use of the principle of predictive breeding in the pot seedling or single hill stage where genotyping is not recommended due to high costs.

### Supplementary Information

Below is the link to the electronic supplementary material.

Supplementary file 1 (pdf 1785 KB)

## Data Availability

The original data sets generated and/or analyzed in the current study are not publicly available due to the material being part of the company secret of SaKa Pflanzenzucht GmbH & Co. KG. However, the data are available in encoded form from the corresponding author upon reasonable request.

## Code availability

R scripts for data analysis are available on GitHub: https://github.com/AlessioMR/ps_in_potato_breeding.
