# Supplementary material for: Using drone-retrieved multispectral data for phenomic selection in potato breeding
Source: Theor Appl Genet. 2024 Mar 6;137(3):70. doi: 10.1007/s00122-024-04567-3 (PMC10917832; doi:10.1007/s00122-024-04567-3)
Supplement: Supplementary file 1 — (pdf 1785 KB) [file 122_2024_4567_MOESM1_ESM.pdf]

SUPPLEMENTARY MATERIAL

Table S1: Overview of spectral data caputred via UAV in each year. Each channel was measured at three different time points per year. The 670nm channel was not assessed in 2021 due to a malfunction of the camera. Different cameras of the same model but with different available channels were used across the years resulting in data of the 570nm channel missing in 2020 and data of the 900nm channel missing in 2021. FWHM stands for full width at half maximum and describes the width of the band around its peak.

Wavelength (nm)	FWHM (nm)	Windeby 2020	Windeby 2021
530	10	data	data
570	10	missing	data
670	10	data	missing
700	10	data	data
730	10	data	data
780	10	data	data
900	20	data	missing

Table S2: Trait explanation and information about statistical procedures for data cleaning.

Trait	Description	Unit	Information
emergence	The time between planting and emergence of plants above ground.	1 - 9	Outliers removed based on residuals and QQ-normal plots.
eye depth	The depth of growing points on the tuber for new plants.	1 - 9	Outliers removed based on residuals and QQ-normal plots.
general impression	Breeder's preference score for suitability for a specific market.	1 - 9	No outliers.
foliage development	The total amount of foliage produced by a plant. Evaluation starts for a whole field when early check clones are flowering.	1 - 9	Outliers removed based on residuals and QQ-normal plots.
foliage development 2	Similar to foliage development. Evaluation is conducted for a whole field when the late check clones are flowering.	1 - 9	No outliers.
yield	Yield per plant in kilograms. Calculated by dividing the total yield of a plot by the number of grown plants in it.	kg	Outliers removed based on residuals and QQ-normal plots. Yield for Windeby 2021 is lower than for the other environments. Unknown reason.
shape long axis	Tuber shape regarding the long axis.	1 - 9	Outliers removed based on residuals and QQ-normal plots.
shape short axis	Tuber shape regarding the short axis.	1 - 9	No outliers.
tuber size	Tuber size.	1 - 9	No outliers.
maturity	Foliage maturity regarding onset of senescence.	1 - 9	Outliers removed based on residuals and QQ-normal plots.
rhizoctonia symptoms	Symptoms of *Rhizoctonia solani* on the tuber surface.	1 - 9	Outliers removed based on residuals and QQ-normal plots.

Table S2 continued

Trait	Description	Unit	Information
skin type	Smoothness of tuber skin.	1 - 4	Outliers removed based on residuals and QQ-normal plots.
scab symptoms	Symptoms of common scab (*Streptomyces scabies*) on the tuber surface.	1 - 9	Outliers removed based on residuals and QQ-normal plots.
starch content	Starch content derived from measuring under water weight of a 5kg tuber sample with an electronic scale.	%	Outliers removed based on residuals and QQ-normal plots.
polyphenol oxidase activity	Enzymatic activity of polyphenol oxidase which is responsible for browning of tubers.	1 - 9	Outliers removed based on residuals and QQ-normal plots.
bruising tolerance	Inclination of the tuber to develop black spots.	%	Outliers removed based on residuals and QQ-normal plots.
texture	Texture of tuber after cooking.	1 - 9	Outliers removed based on residuals and QQ-normal plots.
taste	Taste of the tuber flesh after cooking.	1 - 9	Outliers removed based on residuals and QQ-normal plots.
discolouration after cooking	Degree of greying discoloration of cooked tuber flesh after cooling down for 18-24 hours.	1 - 9	Outliers removed based on residuals and QQ-normal plots.
prop. of small tubers	Proportion of tubers that are smaller than 35 millimeters in diameter.	%	Root transformation applied. Outliers removed based on residuals and QQ-normal plots.
prop. of normal tubers	Proportion of tubers that are bigger than 35 millimeters but smaller than 65 millimeters in diameter.	%	Outliers removed based on residuals and QQ-normal plots.
prop. of large tubers	Proportion of tubers that are bigger than 65 millimeters in diameter.	%	Outliers removed based on residuals and QQ-normal plots.

Table S3: Heritabilities and variance components across all five environments of phenotypic traits and multispectral channel reflectances from model (2). Variance components (σ^2) are given as a proportion of 1, where σ^2_G is the proportion of variance explained by the clone effect, E by environment, $G \times E$ by the clone-environment interaction, B by block, R by row, C by column (if applicable), and ϵ by error. H^2 is the broad-sense heritability of the given trait/channel × flight date combination reflectance. Every value is rounded to the second decimal place.

Trait/Channel	σ^2_G	σ^2_E	$\sigma^2_{G\times E}$	σ^2_B	σ^2_R	σ^2_C	σ^2_ϵ	H^2
starch content	0.69	0.12	0.09	0.01	0.01		0.08	0.93
shape long axis	0.74	0.04	0.12	0.01	0.00		0.09	0.92
bruising tolerance	0.64	0.10	0.19	0.00	0.00		0.06	0.91
prop. of large tubers	0.61	0.08	0.16	0.01	0.01		0.13	0.89
prop. of small tubers	0.60	0.09	0.23	0.00	0.00		0.09	0.88
prop. of normal tubers	0.57	0.07	0.19	0.01	0.01		0.15	0.87
maturity	0.57	0.01	0.30	0.03	0.00	0.01	0.08	0.82
polyphenol oxidase activity	0.51	0.09	0.24	0.01	0.01		0.14	0.81
yield	0.19	0.56	0.11	0.04	0.04		0.07	0.77
emergence	0.33	0.32	0.21	0.01	0.01	0.01	0.11	0.76
rhizoctonia symptoms	0.29	0.38	0.07	0.02	0.00		0.23	0.75
skin type	0.44	0.05	0.42	0.01	0.00	0.01	0.07	0.74
discoloration after cooking	0.39	0.05	0.46	0.01	0.01		0.08	0.74
foliage development 1	0.34	0.20	0.31	0.03	0.02	0.01	0.11	0.71
eye depth	0.37	0.10	0.43	0.01	0.00	0.01	0.08	0.70
tuber size	0.30	0.24	0.11	0.04	0.00	0.02	0.30	0.69
texture	0.29	0.08	0.03	0.01	0.00		0.59	0.65
general impression	0.30	0.02	0.09	0.04	0.00	0.02	0.53	0.60
foliage development 2	0.16	0.48	0.21	0.04	0.00		0.11	0.59
taste	0.19	0.03	0.61	0.01	0.00		0.17	0.49
scab symptoms	0.11	0.39	0.17	0.02	0.05		0.26	0.44
shape short axis	0.11	0.07	0.03	0.01	0.00		0.79	0.30
900nm on D3[b]	0.35	0.04	0.32	0.05	0.03		0.22	0.60
780nm on D3	0.31	0.02	0.39	0.03	0.03		0.23	0.56
730nm on D1	0.20	0.00	0.26	0.23	0.05	0.10	0.16	0.48
730nm on D3	0.23	0.03	0.45	0.05	0.02	0.03	0.18	0.47
570nm on D1[a]	0.26	0.00	0.18	0.13	0.04	0.04	0.36	0.47
530nm on D1	0.25	0.00	0.11	0.11	0.05	0.07	0.41	0.46
780nm on D1	0.20	0.00	0.24	0.24	0.04	0.08	0.19	0.46
700nm on D1	0.21	0.00	0.16	0.12	0.04	0.07	0.40	0.40
530nm on D2	0.15	0.03	0.15	0.17	0.10	0.08	0.31	0.39
670nm on D1[b]	0.20	0.00	0.22	0.10	0.03	0.05	0.40	0.39
900nm on D1[b]	0.18	0.00	0.33	0.16	0.05	0.10	0.18	0.39
570nm on D2[a]	0.16	0.05	0.12	0.17	0.10	0.07	0.33	0.37

Table S3 continued

Trait/Channel	σ^2_G	σ^2_E	$\sigma^2_{G\times E}$	σ^2_B	σ^2_R	σ^2_C	σ^2_ϵ	H^2
570nm on D3[a]	0.12	0.12	0.38	0.12	0.00		0.25	0.37
700nm on D2	0.13	0.04	0.11	0.18	0.09	0.05	0.40	0.31
730nm on D2	0.07	0.02	0.31	0.10	0.19		0.32	0.18
700nm on D3	0.08	0.04	0.53	0.09	0.02		0.25	0.17
530nm on D3	0.07	0.02	0.45	0.13	0.00		0.33	0.14
670nm on D2[b]	0.05	0.00	0.06	0.14	0.03	0.07	0.65	0.13
780nm on D2	0.04	0.02	0.30	0.16	0.14		0.35	0.11
670nm on D3[b]	0.00	0.05	0.63	0.03	0.05	0.05	0.20	0.00
900nm on D2[b]	0.00	0.17	0.19	0.15	0.09		0.41	0.00

[a] Imputed channel: Missing for Windeby 2020
[b] Imputed channel: Missing for Windeby 2021

Table S4: Heritabilities and variance components of multispectral channel reflectances across flight dates and environments from model (5). Variance components (σ^2) are given as a proportion of 1, where σ^2_G is the proportion of variance explained by the clone effect, E by environment, $G \times E$ by the clone-environment interaction, B by block, R by row, C by column, F by flight date, and ϵ by error. The flight date and its interaction terms were of primary interest. H^2 is the broad-sense heritability of the given channel reflectance calculated with model (4) while H^2_{plot} is the heritability on a plot basis. Every value is rounded to the second decimal place.

Channel	σ^2_G	σ^2_E	$\sigma^2_{G\times E}$	σ^2_B	σ^2_R	σ^2_C	σ^2_F	$\sigma^2_{G\times F}$	$\sigma^2_{F\times E}$	$\sigma^2_{G\times F\times E}$	$\sigma^2_{B\times F}$	$\sigma^2_{R\times F}$	$\sigma^2_{C\times F}$	σ^2_ϵ	H^2	H^2_{plot}
530nm	0.10	0.01	0.04	0.04	0.00	0.00	0.00	0.07	0.00	0.18	0.09	0.04	0.03	0.37	0.41	0.20
570nm[a]	0.09	0.02	0.09	0.03	0.00	0.00	0.02	0.12	0.00	0.10	0.12	0.05	0.02	0.34	0.35	0.17
670nm[b]	0.04	0.00	0.11	0.06	0.02	0.03	0.03	0.05	0.00	0.11	0.05	0.02	0.02	0.46	0.18	0.07
700nm	0.05	0.02	0.05	0.05	0.00	0.00	0.00	0.09	0.00	0.21	0.11	0.05	0.03	0.36	0.24	0.11
730nm	0.03	0.00	0.09	0.07	0.00	0.00	0.01	0.15	0.00	0.24	0.08	0.07	0.04	0.22	0.16	0.09
780nm	0.03	0.00	0.11	0.08	0.00	0.00	0.01	0.15	0.00	0.21	0.08	0.06	0.01	0.25	0.14	0.08
900nm[b]	0.00	0.06	0.03	0.09	0.01	0.01	0.00	0.19	0.00	0.26	0.03	0.04	0.03	0.26	0.00	0.00

[a] Imputed channel: Missing for Windeby 2020
[b] Imputed channel: Missing for Windeby 2021

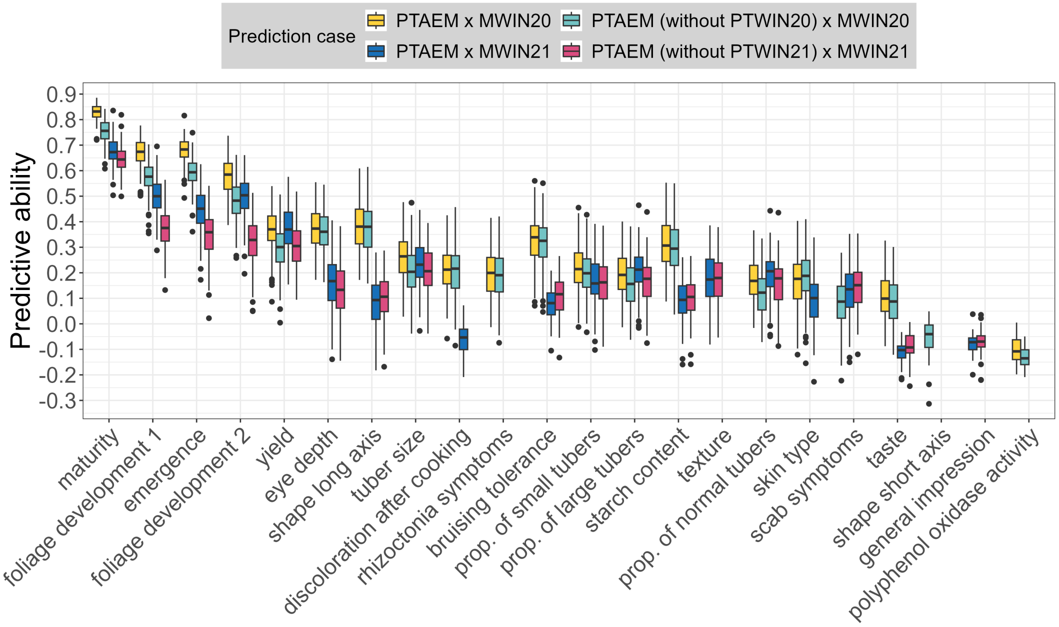

Figure S1: Predictive abilities for scenarios 3 and 3 b. In scenario 3, the MBLUP model was trained with the phenotypic adjusted entry means across all five environments (PTAEM) and a relationship matrix calculated with multispectral data of either Windeby 2020 (MWIN20) or Windeby 2021 (MWIN21). In scenario 3b, AEMs were predicted where the original environment which was the source of the spectral data needed for the relationship matrix was not included in the calculation of the AEMs.

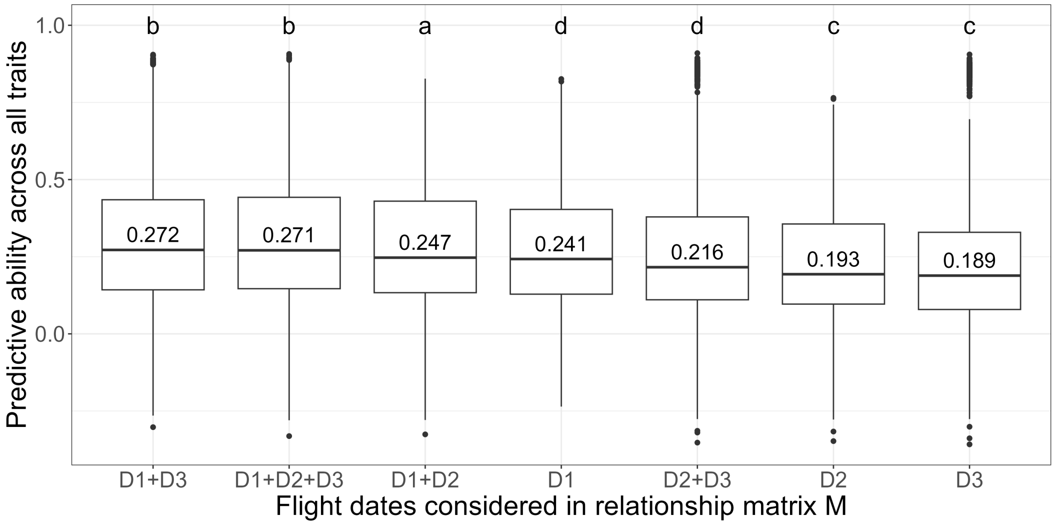

Figure S2: Predictive abilities across all 22 potato traits of adjusted entry means across all five environments considering different combinations of flight dates in the calculation of the relationship matrix M. The letters mark flight date combination pairs that are significantly (p = 0.05) different from each other calculated via Tukey test.

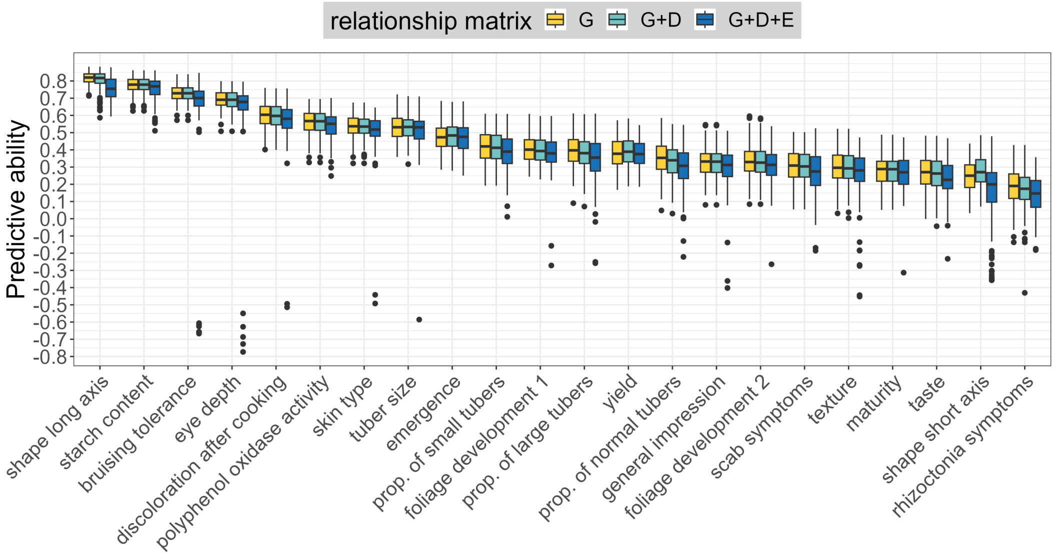

Figure S3: Predictive abilities of predictions including different genetic interaction effects of scenario seven in which adjusted entry means across all five environments were predicted with GBLUP. G, D, and E represent additive, dominance, and epistatic effects, respectively. Every first-degree epistatic interaction effect was considered in G+D+E, namely, additive × additive, additive × dominance, and dominance × dominance.

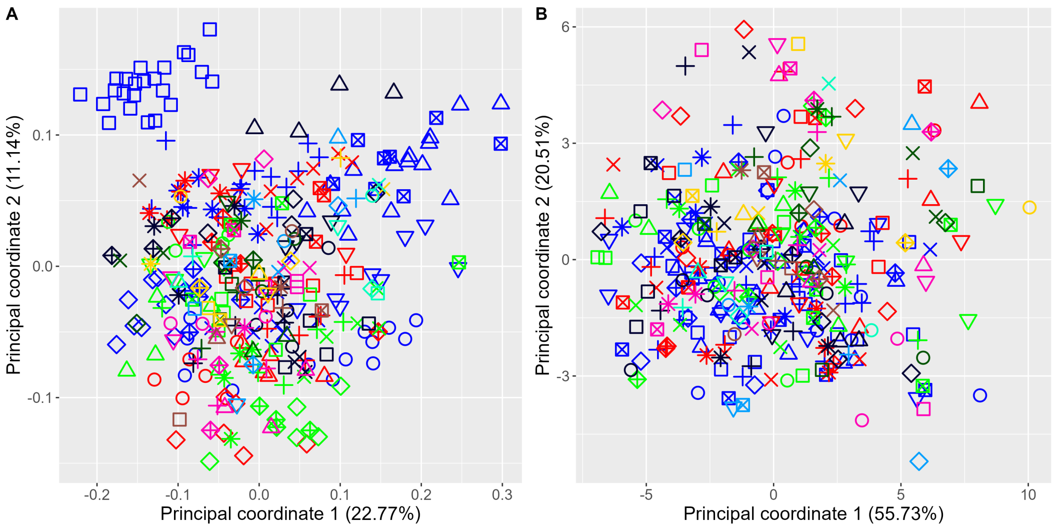

Figure S4: Principal coordinates analysis of our potato population derived either from the genetic relationship matrix (A) or the multispectral reflectance relationship matrix (B). Each colour and shape combination represents a distinct family.
